# Typical Soil Redox Processes in Pentachlorophenol Polluted Soil Following Biochar Addition

**DOI:** 10.3389/fmicb.2018.00579

**Published:** 2018-03-27

**Authors:** Min Zhu, Lujun Zhang, Liwei Zheng, Ying Zhuo, Jianming Xu, Yan He

**Affiliations:** ^1^Institute of Soil and Water Resources and Environmental Science, College of Environmental and Resource Sciences, Zhejiang University, Hangzhou, China; ^2^Zhejiang Provincial Key Laboratory of Agricultural Resources and Environment, Hangzhou, China

**Keywords:** biochar, PCP dechlorination, dissimilatory iron reduction, sulfate reduction, AQDS, molybdate

## Abstract

Reductive dechlorination is the primary pathway for environmental removal of pentachlorophenol (PCP) in soil under anaerobic condition. This process has been verified to be coupled with other soil redox processes of typical biogenic elements such as carbon, iron and sulfur. Meanwhile, biochar has received increasing interest in its potential for remediation of contaminated soil, with the effect seldom investigated under anaerobic environment. In this study, a 120-day anaerobic incubation experiment was conducted to investigate the effects of biochar on soil redox processes and thereby the reductive dechlorination of PCP under anaerobic condition. Biochar addition (1%, w/w) enhanced the dissimilatory iron reduction and sulfate reduction while simultaneously decreased the PCP reduction significantly. Instead, the production of methane was not affected by biochar. Interestingly, however, PCP reduction was promoted by biochar when microbial sulfate reduction was suppressed by addition of typical inhibitor molybdate. Together with Illumina sequencing data regarding analysis of soil bacteria and archaea responses, our results suggest that under anaerobic condition, the main competition mechanisms of these typical soil redox processes on the reductive dechlorination of PCP may be different in the presence of biochar. In particularly, the effect of biochar on sulfate reduction process is mainly through promoting the growth of sulfate reducer (*Desulfobulbaceae* and *Desulfobacteraceae*) but not as an electron shuttle. With the supplementary addition of molybdate, biochar application is suggested as an improved strategy for a better remediation results by coordinating the interaction between dechlorination and its coupled soil redox processes, with minimum production of toxic sulfur reducing substances and relatively small emission of greenhouse gas (CH_4_) while maximum removal of PCP.

## Introduction

Pentachlorophenol (PCP, C6Cl5OH) was first produced in 1930s and extensively used in the following decades until it has been banned globally since late 20th century (Hong et al., 2005; Gao et al., 2008; Ruder and Yiin, 2011). As a representative compound with stable aromatic ring structure and high chlorine content, PCP has relative persistence, high toxicity and long half-life in the natural environment (Zheng et al., 2011; Guyton et al., 2016; Piskorska-Pliszczynska et al., 2016; Louis et al., 2017). Therefore, soils and sediments became the major environmental sinks for PCP as well as its byproducts and were also potential sources of re-emission (Zheng et al., 2012; Chen et al., 2016; Diagboya et al., 2016; Cui et al., 2017). Under anaerobic conditions, reductive dechlorination process has proved to be of paramount importance for PCP degradation and in which PCP acts as an electron acceptor with electrons flow from electron donors. Our previous study had showed that the coexisting ionic species in the flooded soil, such as Fe(III), and SO_4_^2-^, can also be served as terminal electron acceptors during anaerobic redox reactions to compete with PCP (Lin et al., 2014, 2018; Xu et al., 2015; Xue et al., 2017). But as two sides of the same coin, the processes of dissimilation iron and/or sulfate reduction were also found to have positive effect on PCP reduction process mediating by the functional microorganisms and mediators (Ehlers and Rose, 2006; Yang et al., 2009; Xu et al., 2014). The presence of the right terminal electron acceptor is vital for the organohalide respiration process, but it is hard to know the practical effect in the natural environment (Adrian and Loffler, 2016). This makes a more complicated and confused processes for PCP anaerobic degradation in flooded soil. Hence, there is still a lack of understanding of how these natural soil redox process effect PCP dechlorination and the direct or indirect mechanisms involved under anaerobic environment.

Biochar, a carbonaceous material formed during pyrolysis of biomass, is considered as a strong and effective sorbent for contaminated soil remediation (Xiao and Pignatello, 2015; Moreira et al., 2017). It can potentially effect pollutant bioavailability, and modify soil microbial habitats and (or) directly influencing microbial metabolisms, which together induce changes in microbial activity and microbial community structures (García-Delgado et al., 2015; Dai et al., 2016; Yao et al., 2017; Zhu et al., 2017). In addition to their high sorption ability, it has been demonstrated recently that some of these effects on soil biogeochemistry are a direct consequence of its electrochemical properties. Biochars from various feedstock sources can either accept, donate or mediate substantial amounts of electrons in their environment, via abiotic or microbial processes (Prévoteau et al., 2016; Chacón et al., 2017; Yuan et al., 2017). Previous studies have shown that biochar can influence the Fe redox cycling not only indirectly by changing the soil structure and chemistry but also directly by mediating electron transfer processes, i.e., though functioning as an electron shuttle (Kappler et al., 2014; Xu et al., 2016). However, how biochar affects sulfate redox cycling through its modification for sulfate reducer and whether it can act as an electron shuttle during this process are currently unknown.

Moreover, the redox properties of biochar has also been studied and proposed as a possible cause for PCP transformation by enhancing the extracellular electron transfer in soils (Tong et al., 2014; Yu et al., 2015). But these studies were either conducted under relatively ideal circumstances (artificial buffer or optimal reaction conditions) or under the bacterium suspension system without soil, with adequate carbon sources. Further investigation with experimental condition closer to a real natural flooding environment is thus necessary.

In this study, in order to disclose the effects and mechanisms of biochar on soil microorganisms and transformation of PCP that coupled with soil biogeochemical processes under natural flooded soil, and the role of biochar involved in microbial mediated reduction processes, including dissimilatory iron and sulfate reduction, PCP dechlorination, and methanogenesis, were simultaneously investigated. Sterilized controls were set to deduct the changes of environmental physical-chemical processes. A typical electron shuttle, 2,6-sodium anthraquinone disulfonate (AQDS) was added for the comparison of differences in the redox properties of biochar. To determine the mechanisms of biochar effect on sulfate reduction process, molybdate was added as a microbial sulfate reduction inhibitor. We hypothesized that: (1) biochar will promote both ferric iron reduction process and sulfate reduction process, but mechanisms involved may be different; (2) with modification in natural soil redox processes and soil microbial diversity following biochar amendment, biochar’s effect on PCP removal in flooded soil might be very different and complicate with previous found in dryland soil.

## Materials and Methods

### Chemicals

Pentachlorophenol and its degradation intermediates (>98% purity), including 2,3,4,5-Tetrachlorophenol (2,3,4,5-TCP), 3,4,5-trichlorophenol (3,4,5-TCP) and 3,5-Dichlorophenol (3,5-DCP), were purchased from Sigma-Aldrich (St. Louis, MO, United States). The extractants (>99.9% purity), including methanol, n-hexane and acetone, were obtained from Merck KGaA (Darmstadt, Germany). The other analytical grade chemicals were obtained from Sinopharm Chemical Reagent Co., Ltd., Shanghai, China. Anhydrous sodium sulfate was muffle furnace-dried at 750°C for 4 h before use.

### Soil Sampling

A deep layer (80–100 cm) of a coastal mangrove soil was collected near the Taishan city in Guangdong province, China (21°48.991’N, 112°27.848’E). The soil was air-dried, gently ground, and then partly passed through a 1 mm sieve for incubation. The soil had an average pH of 8.9, an organic matter content of 1.16%, and a composition of 17.49% clay, 62.62% silt, and 19.89% sand. The soil sulfate (SO_4_^2-^) and total Fe content were comparatively high and the values of which were 626.3 μg g^-1^ and 33631.7 μg g^-1^, respectively. The other basic physicochemical properties of the soil were analyzed and the results are described in Supplementary Table S1 in the supporting information (SI).

### Biochar Preparation and Characterization

Maize straw biochar was produced from an oxygen-limited muffle furnace at 500°C for 2 h as previously described (Luo et al., 2011). After cooling down to room temperature, the charred materials were milled to approximately 0.15 mm and sieved through a 100-mesh sifter. The elemental C, N, H, and S compositions of the biochar were determined using an elemental analyzer (Vario EL Cube, Elementar Co., Germany), and the O content was estimated by mass balance. The Brunauer–Emmett–Teller (BET) specific surface area of biochar was measured using Mastersizer 3000 (Malvern, United Kingdom). Nuclear magnetic resonance (NMR) analysis of biochar was conducted in the Center of Modern Analysis, Nanjing University (Bruker DRX 500, Germany). The essential properties of the biochar are given in Supplementary Table S2 in SI.

### Anaerobic Incubation Experiment

Each serum bottles (150 mL) contained 15 g air-dry soil, amended with biochar at application levels of 0 and 1% (w/w), respectively. To obtain a PCP-spiked soil with a concentration of 20 μg g^-1^ and maintain a 1:2 (w/v) soil/water mixture to guarantee the flooding condition, 30 mL sterilized Milli-Q water and 0.1 mL PCP stock solution (3000 mg PCP L^-1^, dissolved in acetone) was added to each bottle. The abiotic controls that contained same soil and biochar were sterilized by γ-irradiation at 50 KGy to quantify the loss of PCP due to abiotic processes and systematic loss. For comparison, 100 μM anthraquinone-2,6- disulfonate (AQDS) was added as a known electron shuttle in the non-sterilized soil (Kappler and Haderlein, 2003). To investigate the effect of sulfate reduction process on the reductive transformation of PCP, additional vials also received 20 mM molybdate to inhibit the microbial activities of sulfate reducer (Patidar and Tare, 2005; Aguilar-Barajas et al., 2011). Briefly, three treatment groups were set as: sterilized abiotic group, unsterilized biotic group and unsterilized biotic molybdate group. Each group included three treatments, namely control, AQDS amendement, 1% biochar amendement. The bottles were then followed by vigorous shaking and purged with N_2_ (99.99%) for 20 min (0.75 L min^-1^) to eliminate the O_2_ and the solvent acetone from the experimental systems according to a preliminary study. After then the bottles were sealed with Teflon-coated butyl rubber stoppers and crimp seals. All treatments were incubated at 25°C in an anaerobic chamber (Don Whitley Scientific, England) under a N_2_ stream in the dark, for up to 120 days.

According to our previous study, triplicate samples from each treatment were destructively sampled for analysis at the end of the 120-day incubation for analysis. The sampling procedure was as follows: firstly, the gases (CO_2_, CH_4_) of each bottle was collected by the injection syringe and then injected in a 7 ml vacuum flask. Secondly, the redox potential (Eh) of the soil was measured *in situ* with a platinum electrode and a standard calomel electrode. The pH was also measured *in situ* with a complex electrode. The 0.5 ml slurry was then used for extraction to determine the HCl-extractable Fe(II) after vortexed 2 min. Finally, about two-thirds of the incubation mixtures were sampled and vacuum freezing-dried for other environmental variables (SO_4_^2-^, NO_3_^-^, dissolved organic carbon (DOC), dissolved organic nitrogen (DON), PCP and its intermediates products) analysis, and the remaining slurry was sampled and stored at -80°C immediately for DNA extraction, amplification and high throughput sequencing.

### Analytical Methods

#### Soil Chemical Analysis

The major chemical molecules and ions in soil mentioned above were measured following previously described methods (Xu et al., 2015). Briefly, Fe(II) concentration was measured based on 1,10-phenanthroline spectrophotometer colorimetric method after 0.5 mol L^-1^ HCl extraction for 24 h. Concentrations of anions (SO_4_^2-^ and NO_3_^-^) and DOC/DON concentrations were determined through Milli-Q water extraction at ratio of 1:10 (w/v) before analysis by ion chromatography and TOC analyzer, respectively. The concentrations of PCP and its intermediate products (2,3,4,5-TeCP, 3,4,5-TCP, and 3,5-DCP) in soils were extracted by ultrasonic extraction and subsequent derivatization by mixing with K_2_CO_3_ (10 mL, 0.2 M) and acetic anhydride (0.5 mL) (Lin et al., 2014). A gas chromatograph (Agilent 6890N, Agilent, Santa Clara, CA, United States) equipped with a ^63^Ni electric capture detector (Hewlett-Packard 6890, Hewlett-Packard, Palo Alto, CA, United States) and a HP-5 MS capillary column (30 m by 0.32 mm diameter by 0.25 μm) (J&W Scientific, Inc., Folsom, CA, United States) was used to determine the quantity and the species of chlorophenols. The concentration of released CO_2_ and CH_4_ were monitored by gas chromatography (GC) equipped with a flame ionization detector (FID) combined with a methane converter (TECHCOMP, China).

#### Soil DNA Extraction and Illumina Sequencing

Total microbial genomic DNA was extracted from 0.5 g of soil sample using the MoBio PowerSoil DNA Isolation Kit (MoBio Laboratories, Carlsbad, CA, United States) according to the manufacturer’s instructions. The quantity and quality of extracted DNA were checked photometrically using a NanoDrop^®^ ND-1000 UVeVis spectrophotometer (NanoDrop Technologies, Wilmington, DE, United States). The V4 region of the bacterial 16S rRNA gene was amplified by the polymerase chain reaction (PCR) with the primer pair 520F (5′-AYTGGGYDTAAAGNG-3′) and 802R (5′-TAGNVGGGTATCTAATCC -3′). For archaeal genes, the V5-V6 region was amplified by the PCR. The forward and reverse primers were U789F: 5′-TAGATACCCSSGTAGTCC-3′ and U1068R: 5′-CTGACGRCGCCATGC-3′, respectively. The procedures for bacterial and archaeal DNA amplification were conducted by Personal Bio Co., Ltd., Shanghai, China. The sequences were submitted to the NCBI Sequence Read Archive (SRA) database (with accession number SRP127655 for the bacteria and SRP127707 for the archaea).

### Statistical Analysis

Statistical analyses were performed using SPSS software version 18.0 (SPSS, Chicago, IL, United States). Treatment effects were tested by oneway ANOVA. Statistical significance was determined at the 5% level. To compare soil microbial communities, non-metric multidimensional scaling (NMDS) was given based on generalized UniFrac distance with “vegan” and “GUniFrac” packages on R platform^1^. UniFrac distance measured phylogenetic dissimilarities between communities (Lozupone and Knight, 2005) and NMDS visualized the distance in low dimensional space. Parameter α was set at 0.5 when calculating generalized UniFrac and 4 axes were remained for both archaea and bacteria to reduce stress less than 0.05. Dissimilarities between treatments were tested using permutation multivariate analysis of variance (PERMANOVA) (Anderson, 2001). Only the main effects of conditions (with or with molybdate) were tested. Associations between dominant species with abundance greater than 1% in archaea or bacteria and environmental factor were analyzed following Spearman’s method and visualized using “ggplot2” package. The raw *p*-values were adjusted following Benjamini and Hochberg’s (1995) procedure and the adjusted *p*-value less than 0.05 was considered significant.

## Results

### Concentrations of PCP Residuals and Dechlorination Products

**FIGURE 1:**
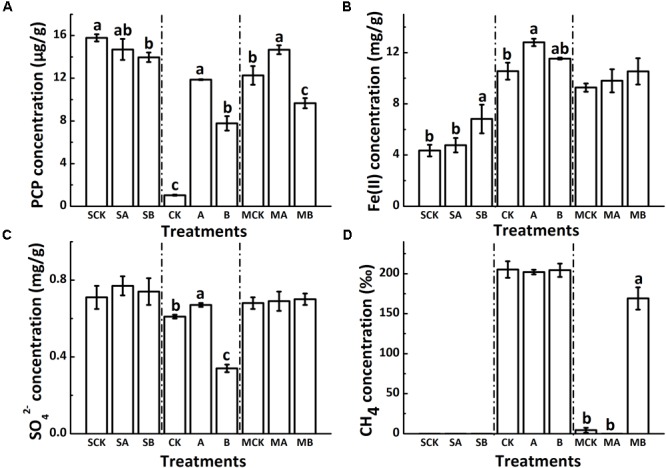
Different soil redox processes at 120 days as shown by the concentration of PCP, Fe(II), SO_4_^2-^ and CH_4_ **(A–D)**. CK: Soil + none; A: Soil + AQDS; B: Soil + 1% biochar; the prefix “S”: sterilized abiotic treatment group; the prefix “M”: unsterilized biotic molybdate treatment group. Only significant differences (*p* < 0.05) were shown by the letter (a, ab, b, c) in lowercase on the top of data column.

The residual concentrations of PCP in soils of all treatments are shown in **Figure 1A**. The PCP dissipation extents in biotic treatments were obviously greater than those in abiotic treatments. For the abiotic control treatment, PCP decreased from the initial value of 20 μg g^-1^ to 15.8 μg g^-1^ after 120 days. AQDS addition did not significantly affect the abiotic removal of PCP (14.7 μg g^-1^); but biochar significantly decreased the residual concentration of PCP (13.9 μg g^-1^) (*p* < 0.05, similarly hereinafter). For the biotic treatment groups, the degradation extent of PCP was significantly decreased by an average of 40% in the molybdate amended treatments. PCP degradation was significantly restrained with AQDS or biochar, especially with AQDS. Regardless of amendment molybdate, the PCP degradation extent decreased in the following order: soil + none (CK) > soil + 1% biochar (B) > soil + AQDS (A).

The major detectable intermediate products in the biotic treatment groups during PCP dechlorination were 2,3,4,5-TeCP and 3,4,5-TCP (Supplementary Figure S1), which were not detected in the abiotic treatment group. The variation of 2,3,4,5-TeCP concentration in different treatments was in accordance with PCP degradation extent, while 3,4,5-TCP stayed at very low concentrations (about 0.5 μg g^-1^) in all treatments. Comparing with biochar-free controls, biochar did not change the 2,3,4,5-TeCP concentration significantly, with the final concentration of about 5 μg g^-1^ and 10 μg g^-1^ in the presence and absence of molybdate, respectively. Among all the biotic treatments, soils with AQDS accumulated the minimum amount of 2,3,4,5-TeCP both in the presence or absence of molybdate, which were 1.2 μg g^-1^ and 4.5 μg g^-1^, respectively.

### Changes of Typical Redox Processes

The concentration of reduced iron [Fe(II)] (**Figure 1B**) was comparatively small in the abiotic group (ranging from 4.4 mg g^-1^ to 6.8 mg g^-1^, close to the natural background levels of the soil samples (about 3 mg g^-1^). In the biotic treatment groups, the increased concentration of Fe(II) multiplied and the average concentration of which was about 10 mg g^-1^. Compared with biochar-free controls, biochar increased the accumulation of Fe(II), with changes not significant in all biotic treatments. In the absence of molybdate, the concentration of Fe(II) significantly increased by AQDS addition and reached a maximum values of 12.8 mg g^-1^ among the biotic treatments.

The sulfate concentration in soils decreased slightly in the biotic treatments with molybdate (on an average of 0.69 mg g^-1^) as compared to that in the abiotic controls (on an average of 0.74 mg g^-1^) (**Figure 1C**). Regardless of sterilization, neither AQDS nor biochar had significant effects on sulfate reduction in the treatments with molybdate. However, in the biotic treatments without molybdate, the sulfate reduction was significantly increased by biochar (from 0.74 to 0.34 mg g^-1^) but decreased by AQDS (from 0.77 to 0.67 mg g^-1^), as compared to that in the biochar-free control.

No methane was released from the soils of all abiotic treatments (**Figure 1D**). For the biotic treatments, the methanogenesis process was also inhibited with molybdate addition, with the concentration of methane only 4.34‰ in the control vials. Amendment of both molybdate and AQDS even further suppressed the release of methane to an undetectable level. However, the methanogenic activities could be increased with the coexistence of biochar, and the concentration of methane reached 169.1‰. For the biotic treatments without molybdate, the methanogenesis process was fully conducted and the concentration of methane reached a maximum of approximately 200‰, with no significant differences detected among this treatment group.

### Changes of Bacteria and Archaea Communities

**FIGURE 2:**
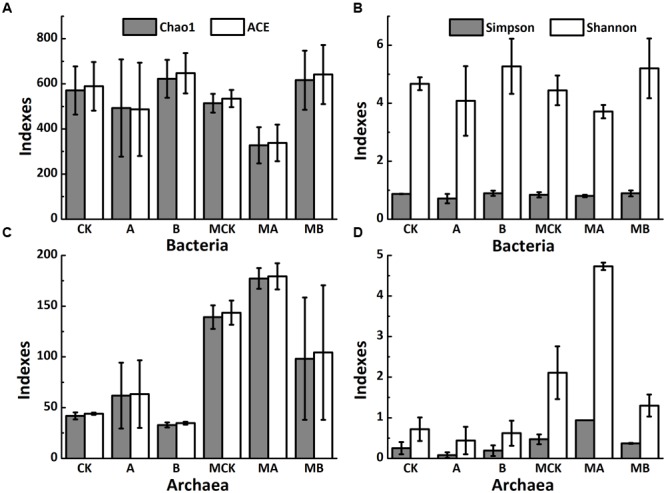
Alpha diversity indexes of soil bacteria **(A,B)** and archaea **(C,D)** of different treatments. Abbreviations of the treatments are as **Figure 1**.

Taxonomic identity of each phylotype was determined using the Greengenes Classifier. A total of 777,455 and 818,579 trimmed sequences with the length of > 150 bp were obtained, and 1,600 and 389 operational taxonomic units (OTUs) with 97% similarity were identified for bacteria and archaea, respectively, from 18 soil samples of the biotic treatments. **Figures 2A,B** shows that the abundance-base diversity (α-diversity) indices of ACE, Chao 1, Simpson and Shannon of bacteria increased with biochar amendment but decreased with AQDS amendment in the presence or absence of molybdate. Comparing to the non-molybdate treatments, these indexes value slightly decreased with molybdate amendment. Besides, the changes of the α-diversity of archaea were exactly the opposite (**Figures 2C,D**). Molybdate addition increased α-diversity of archaea, these four indexes significantly increased in the AQDS treatments but decreased in the biochar treatments.

**FIGURE 3:**
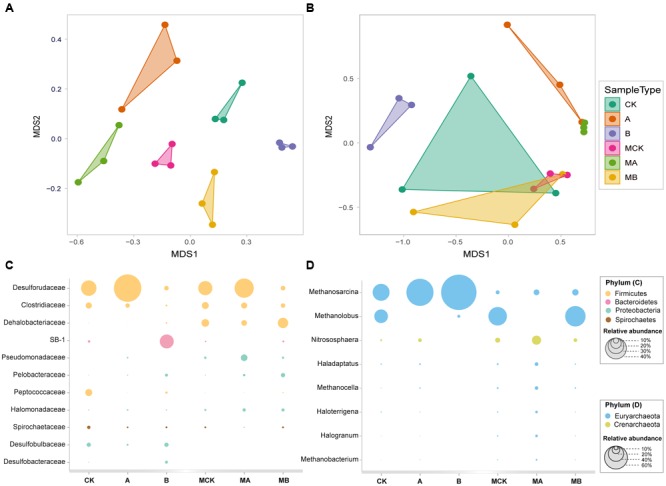
The NMDS plots based on generalized UniFrac distance for bacterial **(A)** and archaeal **(B)** community structure, respectively, and the relative abundance of dominant bacterial community at family level **(C)** and archaeal community at genus level **(D)** in different treatments.

The non-metric multi-dimensional scaling (NMDS) analysis of OTUs relative abundance of bacteria (**Figure 3A**) showed an obvious separation of the six biotic treatments (stress = 0.04), indicating a significant effect of AQDS and biochar on bacteria communities along the MDS1, with the molybdate treatments obviously separated from the three control treatments along the MDS2. Additionally, the two treatments with AQDS, and the treatment with biochar only were also clearly separated from the other tree treatments in **Figure 3B** (stress = 0.03). This also indicated significant differences of these treatments in archaeal communities. The other two dimension (MDS3 and MDS4) plot of bacteria and archaea were plotted in Supplementary Figure S2.

Bacterial relative abundance (>2%) of the six treatments was compared at family level (**Figure 3C**). Treatment groups with or without molybdate had the similar variation among the control, AQDS and biochar treatments. The most abundant phylum in the control was *Firmicutes* (>60%), followed by *Bacteroidetes* (>16%), *Proteobacteria* (>15%) and *Spirochaetes* (>3%). Compared with the control, the addition of biochar increased the relative abundances of *SB-1, Dehalobacteriaceae, Pelobacteraceae, Desulfobulbaceae*, and *Desulfobacteraceae* significantly (especially the *SB-1* that increased from 4.5 to 26.5%), but decreased the relative abundances of *Clostridiaceae* and *Peptococcaceae* significantly. With the amendment of AQDS, the relative abundance of *Desulforudaceae* increased significantly from 29.3 to 52.3%. The relative abundances of *Pseudomonadaceae* and *Halomonadaceae* also increased in the AQDS treatment. In the presence of molybdate, the relative abundance of *Desulforudaceae, Peptococcaceae, Spirochaetaceae, Desulfobulbaceae*, and *Desulfobacteraceae* decreased significantly, while that of *Clostridiaceae, Dehalobacteriaceae, Pseudomonadaceae*, and *Halomonadaceae* had a significant increase.

The archaea was mainly dominated by the phylum of *Euryarchaeota*, whose relative abundance accounted for > 90% of the control. Archaeal relative abundance (>1%) at genus level was plotted in **Figure 3D**. *Methanosarcina* and *Methanolobus* were the dominant genera, with their relative abundances accounted for 43.9 and 35.3% in the control, respectively. Biochar and AQDS amendment significantly increased the relative abundance of *Methanosarcina* to 92.1 and 70.5%, respectively. Besides, the relative abundance of *Methanolobus* significantly decreased to 7.4 and 0.01% in the biochar and AQDS treatments, respectively. In the presence of molybdate, the relative abundance of *Methanosarcina* of these treatments significantly decreased to no more than 20%, while the relative abundance of *Methanolobus* increased significantly except in the AQDS treatment. The other archaea genera were all increased in the coexistence of molybdate and AQDS, only with the exception of *Methanosarcina* and *Methanolobus*.

### Correlations of Environmental Variables and Microbial Taxonomies

**FIGURE 4:**
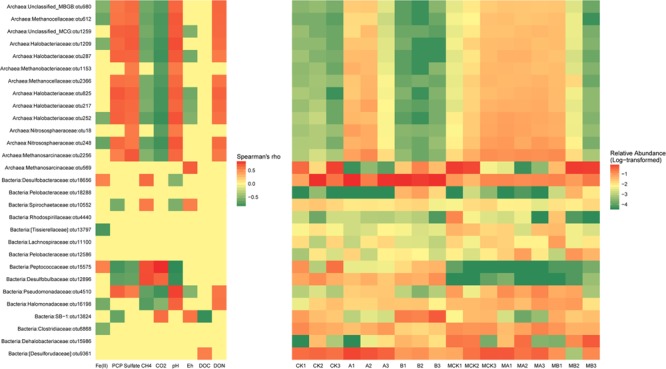
The significant correlations (*p*-value < 0.05) between environmental factors and relative abundance of the dominant bacterial and archaeal OTUs (Left) and the relative abundance of these OTUs in different treatments (Right).

Heatmap based on the relative abundances of the dominant OTUs (>1%) in the data sets as gave detailed classification information between different treatments. As shown in **Figure 4**, all the dominant OTUs of archaea except OTU569, had exactly the same correlations with the environmental variables, which were positively correlated with the concentration of PCP, sulfate, DON and soil pH, but negatively correlated with the concentration of Fe(II), CH_4_, CO_2_, and soil Eh. The relative abundance of these OTUs increased in the presence of AQDS and/or molybdate. The outstanding OTU569 that belongs to family *Methnosarcinaceae* decreased significantly in the AQDS treatments. As to the dominant OTUs of bacteria, their relationship with environmental variables was more changeable. The OTU18656 (*Desulfobacteraceae*) and OTU15575 (*Peptococcaceae*) were positively correlated with the Fe(II) and CH_4_. The relative abundance of these two OTUs increased in the treatments with AQDS or biochar. The OTU10552 (*Spirochaetaceae*), OTU15575 (*Peptococcaceae*), and OTU12896 (*Desulfobulbaceae*) had negative correlations with PCP residues and soil pH, but positively correlated with CH_4_. The relative abundance of these three OTUs increased in the absence of molybdate. The OTU13824 (*SB-1*) also had a negative correlation with PCP residues, but its relative abundance increased in the presence of biochar.

## Discussion

Many studies have indicated that biochar amendment can directly and indirectly affect the fate of persistent organic pollutants and pesticides by acting as a geosorbent (Sun et al., 2012; Anyika et al., 2015), so the γ-irradiation sterilized abiotic treatment group was set in this study, in which the decreases in PCP concentration might be mainly due to sorption contribution of biochar. However, as no chlorophenols metabolites were detected in the sterilized abiotic soils, the sorption amount by biochar could not be deducted through comparison of differences between the abiotic control group and the biotic treatment group. Anyway, the results demonstrated that only a small proportion of PCP (less than 10%) was absorbed by amended biochar after 120-day incubation. Hence, the specific adsorption capacity and maximum adsorption capacity of biochar were not considered, and we speculated the differences in depletion of PCP and its metabolites among the treatments were mainly caused by the degradation ability of indigenous microorganisms in different treatments. The typical soil redox processes, sulfate reduction and methanogenesis, were also not significantly affected by biochar in the abiotic treatments (**Figure 1**).

### The Role of Biochar in Enhanced Fe(III) Reduction Process

Usually, biochar is considered as a soil conditioner in many studies to improve soil fertility by increasing the pH and nutrient retention and shift soil biological community composition and abundance in soil and sediments (Tong et al., 2014; Yu et al., 2015, 2016). Biochar did not have a significant effect on the soil pH as the initial pH of the experimental soil is alkaline (Supplementary Figure S3A). The HCl-extractable Fe(II) is commonly accumulated as an end product of microbial Fe(III) reduction in natural environments. Therefore, a potential explanation for the enhanced generation of HCl-extractable Fe(II) in the presence of biochar is that biochar potentially stimulated the growth and activity of Fe(III) reducer. Our results showed that the addition of biochar and AQDS led to a significant increase in the abundance of *Pelobacteraceae*, especially in the presence of biochar (**Figure 3C**). This family has been discovered as the dominant iron reducer in many studies (Hori et al., 2015), and reported as having a positive correlation with Fe(III) reduction in the ferrihydrite enrichment with the amendment of AQDS or biochar (Zhou et al., 2016).

Meanwhile, biochar has also been reported as an electron shuttle for microbial electron shuttling to transfer electrons onto a solid Fe(III) (ferrihydrite) electron acceptor from *Shewanella oneidensis* and significantly increased the rate of ferrihydrite reduction and extent of reduction (Kappler et al., 2014; Xu et al., 2016; Yuan et al., 2017). Hence, as biochar can donate, accept, or transfer electrons in their surrounding environments, either abiotically or via biological pathways, there is the possibility that biochar served as an electron mediator to enhance the generation of HCl-extractable Fe(II). As a commonly used quinone containing analog of humic substances in laboratory, AQDS is often used as a model electron shuttling compound in studies of dissimilatory microbial reduction of iron oxides and transformation of reductive organic compounds (Kappler and Haderlein, 2003; Liu et al., 2007; Kwon and Finneran, 2008; Costa et al., 2010). Though the above two pathways might thus work concurrently, given that the more prominent increase of HCl-extractable Fe(II) accumulated in the AQDS treatment, the electron shuttling pathway might play a more significant role in the Fe(III) reduction process with biochar amendment.

### Effect of Biochar on Sulfate Reduction Process

Microbial dissimilatory sulfate reduction is also an important transformation that occurs under anaerobic environment (Muyzer and Stams, 2008). After 120 days incubation, the redox potential became downward into the range of SO_4_^2-^ reduction (approximately-150 mV, Supplementary Figure S3B) (Connell and Patrick, 1968). But comparing with the abiotic group, the microbial reduction extend of sulfate was not strong in soils of the biotic treatments. Theoretically, competitive relationships are involved between different microbial metabolic pathways on account of their corresponding thermodynamic feasibility (Jin and Bethke, 2007; Burgin et al., 2011). As such, the microbial reduction of Fe(III) process is prior to the sulfate reduction process and always acts as a powerful competitor when both processes exist simultaneously. Hence, the results that why the concentration of HCl-extractable Fe(II) reached the maximum while the sulfate was reduced at the smallest extent in the presence of AQDS may be well explained (**Figures 1B,C**). This also provided a further proof that the electrons of this system might not be inadequate to the subsequent redox reactions after iron reduction. Put another way, electron shuttling might be not conducive to the microbial sulfate reduction if the iron reduction process is relatively active, especially under electron limited reducing environment.

The effect of biochar on microbial sulfate reduction process has been little investigated so far. The barely researches reported that biochar amendment did not increase the sorption capacity of soil for SO_4_^2-^ (Zhao et al., 2017) and it could enhance the SO_4_^2-^ reduction (to sulfide) by 85% compared to the initial concentration (Easton et al., 2015), but the mechanisms involved had not been well discussed. In our study, biochar amendment also significantly increased the microbiological reduction of SO_4_^2-^, which is highly consistent with increased abundance of sulfate reducer (*Desulfobulbaceae* and *Desulfobacteraceae*) with biochar amendment (**Figure 3C**). But the relative abundance of family *Desulforudaceae*, which has been observed likely involved in the biogeochemical cycling of sulfur in previous study (Rempfert et al., 2017), decreased significantly in the presence of biochar. We thus speculate that this family might not be the main active sulfate reducer in our study. Actually, the existing reports regarding the sulfate reducing function of this family in complex matrices such as soil is still limited. The molybdate ion is a functional analog sulfate during the process of cellular respiration that can be transported into the bacteria, resulting in the deprivation of sulfur reducing compounds (Patidar and Tare, 2005; Aguilar-Barajas et al., 2011). Thus, it acts as an ion specific metabolic inhibitor that limits sulfate reduction and is toxic to these microorganisms. Here, in the presence of molybdate, the relative abundances of the two families *Desulfobulbaceae* and *Desulfobacteraceae* decreased to almost zero with no significant difference among the three treatments, even in the presence of biochar (**Figure 3C**). Therefore, we deduced that the sulfate reduction process under anaerobic environment is predominantly controlled by the functional sulfate reducer but not the electron mediators like AQDS. The effect of biochar on sulfate reduction process is mainly through modifying the abundance and activities of functional microorganisms but not as an electron shuttle.

### Coupling Effect of Biochar and Soil Redox Processes on PCP Reductive Dechlorination

Conventionally, organic contaminants sorbed onto biochar have been considered to be chemically and biologically inert (Lou et al., 2011; Xiao and Pignatello, 2015). The soil residual concentration of PCP in the biotic treatment with biochar was less than that without biochar (**Figure 1A**), which indicated that the reduced portions might be ascribed to the irreversible adsorption by biochar and this might decrease the microbial availability of PCP. Meanwhile, the experimental biochar in our study was produced at 500°C with comparatively high aromaticity and different surface functional groups (Supplementary Table S1), hence, the possibility that biochar acting as an electron shuttle in anaerobic environment should not be discounted. Additionally, similar to the role of biochar in soil redox processes, biochar also behaved multifunction in the biotransformation of organic contaminants in many studies (Zhu et al., 2017). Though a previous study has reported that biochar could positively enhance the extracellular electron transfer in soils to promote PCP transformation by stimulating the growth and metabolism of microorganisms in the soils, it is not close to the real soil environment by maintaining the pH at 7.0 with 30 mM PIPES buffer (Tong et al., 2014). The natural anaerobic soil environment is always more complicated with different electron donors, electron mediators, acceptors and microorganisms (Xu et al., 2015). In our study, the soil:water ratio was set at 1:2 to simulate the flooding environment and we found that AQDS and biochar suppressed the PCP degradation, especially with the AQDS amendment. The original DOC and DON concentrations of the deep soil layer samples used in this study had been relatively low (about 150 mg kg^-1^ and 15 mg kg^-1^, respectively, as shown in Supplementary Figures S3C,D), and we did not add any extra electron donors during the incubation. Therefore, the electron donors in the experimental soil were speculated to be very limited for guaranteeing a complete soil reduction processes. Under this circumstance, limited electrons might be transferred to the dominant more competitive one (in our case, Fe(III) reduction) by the amendments (AQDS or biochar) and thus inhibited the reductive dechlorination of PCP indirectly.

Though the relative abundance of the family *Dehalobacteriaceae* increased significantly in the presence of molybdate (**Figure 3C**), the PCP degradation extent reduced significantly comparing with the molybdate-free treatments (**Figure 1A**). It is inferred that the family *Dehalobacteriaceae* might be the main PCP dechlorinator in the molybdate-free treatments in this study. However, with the coexistence of molybdate and biochar, the relative abundance of this family is comparatively increased, which is coincidence with the enhanced PCP degradation. So biochar might have the ability to benefit the growth of dechlorinators by improving the environmental condition for the dechlorinators and easing the competition relation between dechlorinators and other microorganisms to affect the dechlorination process in the presence molybdate. Besides, the family *Peptococcaceae*, whose abundance decreased significantly in the presence of molybdate, includes many degrading genera like *Dehalobacter* and *Desulfitobacterium* (Dennie et al., 1998; Kranzioch-Seipel et al., 2016). Molybdate has been reported as capable of partially inhibit the dechlorination of polychlorinated biphenyls at a low concentration (1 mM) (Ye et al., 1999). Thus, it would not rule out the possibility that molybdate could inhibit other potential dechlorinators or microorganisms with other functions (e.g., sulfate reducer) to indirectly regulate the reductive dechlorination of PCP.

**FIGURE 5:**
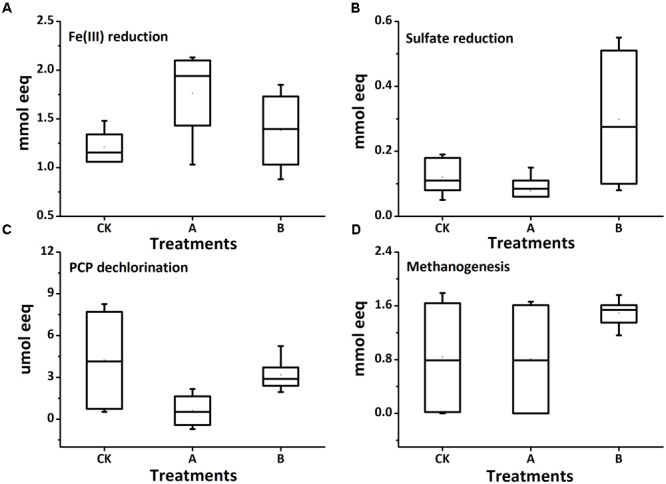
Stoichiometric electron equivalent analysis of the receiving electrons during reduction of Fe(III) **(A)**, SO_4_^2-^**(B)**, PCP **(C)**, and methanogenesis **(D)** in the biotic treatments. Treatments with or without molybdate were grouped together for analysis.

The electrons consumed for each microbial reduction process in biotic treatments were calculated and shown in **Figure 5** (Treatments with or without molybdate were grouped together for analysis; specific values of each treatment were plotted in Supplementary Table S3 in SI). The addition of molybdate decreased the total amount of electron equivalents needed by more than 50% (from an average of 3568 to 1759 μmol) (The calculation of each electron acceptor was based on assumption in **Table 1**). Total electrons consumed by microbial Fe(III) and sulfate reduction processes were significantly increased by the amendment of both AQDS and biochar, respectively, in the molybdate-free treatments [from 1217 to 2096 μmol and from 165 to 501 μmol for Fe(III) and sulfate reduction processes, respectively]; while the electrons subdivided to PCP reduction process were all decreased significantly. This suggested the presence of AQDS or biochar might shift part of electrons from dechlorination to Fe(III) and sulfate reduction. And interestingly, when the whole microbial reduction processes were inhibited to some extent by molybdate, the electrons consumed for dechlorination and methanogenesis significantly increased with biochar addition (from 0.15 to 0.40 μmol and from 34.56 and 1351.80 μmol for dechlorination and methanogenesis, respectively). Therefore, to make sure the exact biochar effect on reductive removal of PCP in flooded soil, more synthetic consideration is necessary to warrant a better result through balancing all the redox processes to avoid the production of both toxic reduced iron/sulfur substances and greenhouse gases while pollution remediation.

**Table 1:** Relevant half reactions of the electron acceptors during 120 days incubation.

Compound	Half Reaction	eeq/mol compound	Reference
Ferrous iron	Fe^3+^ + e^-^ = Fe^2+^	1	Picardal et al., 1995
Sulfides	SO_4_^2-^ + 9H^+^ + 8e^-^ = HS^-^ + 4H_2_O	8^a^	Flynn et al., 2014
Methane	CH_3_COOH = CH_4_ + CO_2_	8^b^	Ferry, 1992;
	CO_2_ + 8H^+^ + 8e^-^ = CH_4_ + 2H_2_O		Liu and Whitman, 2008
TeCP^c^	C_6_Cl_5_OH + H^+^ + 2e^-^ = C_6_HCl_4_OH + Cl^-^	2	Kenneke and Weber, 2003
TCP^d^	C_6_Cl_5_OH + 2H^+^ + 4e^-^ = C_6_H_2_Cl_3_OH + 2Cl^-^	4	

*^*a*^Based on the assumption that the final reduction products during SO_*4*_^*2*-^ reducing process are entirely existed in -2 valence; ^*b*^Calculated with an average electron equivalents that required by one mole methane from acetate metabolism; ^*c,d*^The electron equivalents from PCP transformation were calculated from the concentration changes of the main products of TeCP and TCP that generated from PCP dechlorination.*

### The Potential Functional Microbial Species Regulating Typical Soil Redox Processes in PCP Polluted Soil Following Biochar Addition

Based on the sequencing results, our studies clearly show that the addition of AQDS and biochar had significant influences on the archaea and bacteria structures (**Figures 3A,B**). The corresponding changes of the dominant OTUs (relative abundance > 1%) were analyzed relating to the environmental variables and specific treatments (**Figure 4**). As the most abundant genus in the molybdate-free treatments, genus *Methanosarcina* apparently was the dominant methanogens in these treatments. However, the relative abundance of OTU569 (*Methanosarcina, Methanosarcinaceae*) was irrelevant to CH_4_ but positively related with the DOC. There was no difference with CH_4_ concentration among the treatments, evidently proved that this OTU was susceptible to the readily usable carbon source (electron donors). Another possible explanation is that OTU569 might be very stable in each treatment and had no relations with the major environmental variables.

Positive correlations of PCP with the notable OTU4510 that belongs to the family *Pseudomonadaceae* indicated that this group was resistant to PCP. The dominant OTUs included OTU10552 (*Spirochaeta, Spirochaetaceae*), OTU15575 (*Desulfosporosinus, Peptococcaceae*), OTU12896 (unclassified *Desulfobulbaceae*) and OTU13824 (unclassified *SB-1*) showed negative correlation with PCP residual, indicating these species might participate in the PCP dechlorination. The members of the *Desulfosporosinus* and *Desulfobulbaceae* have been previously suggested as popular sulfate reducer (Miletto et al., 2011; Engelbrektson et al., 2014). Since our results found that both OTU15575 and OTU12896 had a negative correlation with sulfate concentration, they would thus probably be the main functional sulfate reducers. In addition, these two OTUs were positively related to CH_4_ and CO_2_, which might also facilitate the methanogenesis process synergistically by accelerating the reduction of redox potential. Though these two species were found to be important for toluene and hexahydro-1,3,5-trinitro-1,3,5-triazine (RDX) degradation under various electron-accepting conditions (Sun et al., 2012; Cupples, 2016; Michalsen et al., 2016), they have not yet been reported in the chlorinated organic pollutants degradation researches. Their effects on PCP degradation might thus be in an indirect way.

The concentration of accumulated HCl-extractable Fe(II) was positively related to OTU18656 (unclassified *Desulfobacteraceae*) and OTU15575 *(Desulfosporosinus, Peptococcaceae*) whose relative abundances increased in the presence of biochar (**Figure 4**). This indicated that these two species played an important role in facilitating the Fe(III) reduction. It is reported that *Desulfobulbaceae* could partially share the electrons from the benzene as syntrophic partners in an iron-reducing enrichment culture (Kunapuli et al., 2007). Meanwhile, members of the family *Desulfobacteraceae* was also proved to be important for naphthalene degradation under sulfate-reducing conditions in freshwater environments (Kümmel et al., 2015). Therefore, members of this family might also be the muti-functional species that acted as both Fe(III) and SO_4_^2-^ reducer under the stress of PCP pollution, especially in the presence of biochar.

## Author Contributions

MZ designed and carried out the research, data handling and analysis, and wrote the manuscript. LuZ contributed to the analysis of the Illumina sequencing data. LiZ and YZ gave assistance in lab work. JX provided the experimental materials and research platform. YH contributed to the design of the experiments, data mining, and revised the manuscript. All authors read and approved the final manuscript.

## Conflict of Interest Statement

The authors declare that the research was conducted in the absence of any commercial or financial relationships that could be construed as a potential conflict of interest.

## Footnotes

1 http://www.r-project.org
