# Supplementary material for: Typical Soil Redox Processes in Pentachlorophenol Polluted Soil Following Biochar Addition
Source: Front Microbiol. 2018 Mar 27;9:579. doi: 10.3389/fmicb.2018.00579 (PMC5880936; doi:10.3389/fmicb.2018.00579)
Supplement: Supplementary file 1 [file Data_Sheet_1.DOC]

## Supplementary Material

## Typical soil redox processes in pentachlorophenol polluted soil following biochar addition

**Min Zhu**^1,2^**, Lujun Zhang**^1,2^**, Liwei Zheng**^1,2^**, Ying Zhuo**^1,2^**, Jianming Xu**^1,2^**, Yan He**^1,2^,**^*^**

*^1^ Institute of Soil and Water Resources and Environmental Science, College of Environmental and Resource Sciences, Zhejiang University, Hangzhou 310058, China*

*^2^ Zhejiang Provincial Key Laboratory of Agricultural Resources and Environment, Hangzhou 310058, China*

^*^Corresponding author: Yan He; E-mail: yhe2006@zju.edu.cn; Tel.: +86-571-8898-2065; Fax: +86-571-8898-2065

***Correspondence:** Yan He. **Tel.:** +86-571-8898-2065, **Email:** yhe2006@zju.edu.cn

**Detailed information of the Supplementary Material**

The number of Figures: 3

The number of Tables: 3

**Suporting Information**

**Suporting Data**


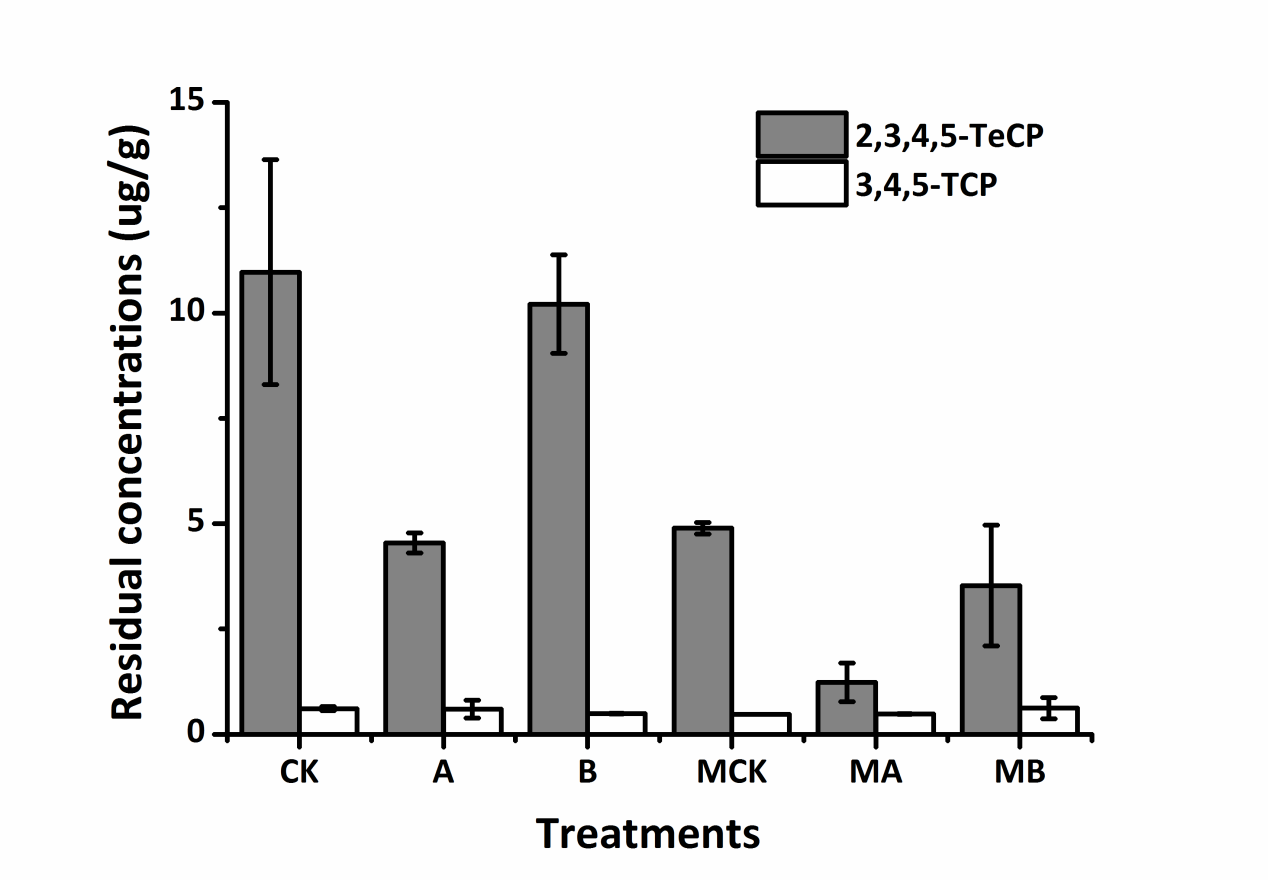
**Figure S1** Residual concentration of the intermediate products of PCP. CK: PCP + none; A: PCP + AQDS; B: PCP + 1% biochar; the prefix “M”: unsterilized biotic molybdate treatment group.


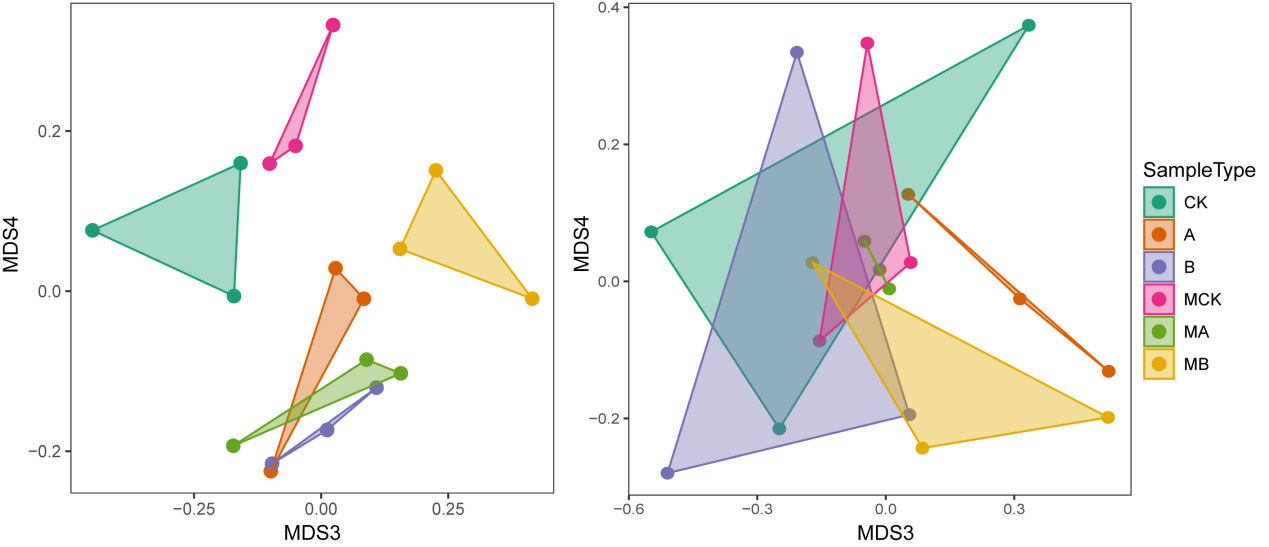
**Figure S2** NMDS analysis (MDS3 and MDS4 axises) in different treatments between environmental factors and bacteria (stress=0.04, A) and archaea (stress=0.03, B) community structure, respectively.


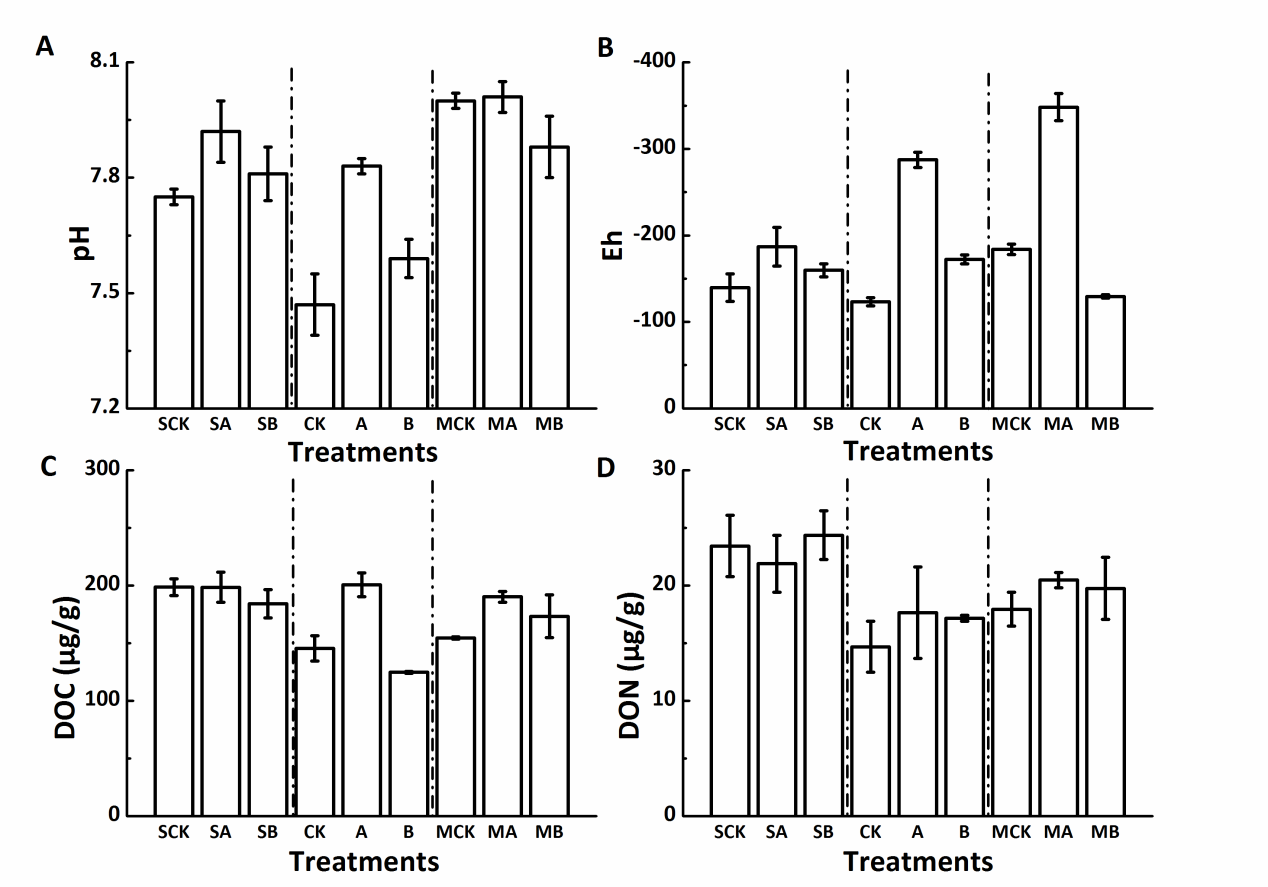
**Figure S3** Effects of biochar amendment on basic properties of soil (A-D). CK: PCP + none; A: PCP + AQDS; B: PCP + 1% biochar; the prefix “S”: sterilized abiotic treatment group; the prefix “M”: unsterilized biotic molybdate treatment group.

**Table S1** Basic properties of experimental soil

| pH | Eh | EC | SO_4_^2-^ | Total Fe | Total Mn(II) | Total Al | Total C | DOC | DON |
| --- | --- | --- | --- | --- | --- | --- | --- | --- | --- |
|  | (mV) | (μs/cm) | mg kg^-1^ | | | | | | |
| 8.9 | 170 | 3346.3 | 626.3 | 33631.7 | 850.4 | 13193.1 | 11903.3 | 133.8 | 9.9 |

**Table S2 Basic properties of experimental biochar**

| pH | BET | Ash |  | Elemental composition (%) | | | | |  | Atomic ratio | |  | NMR parameters (%) | | |
| --- | --- | --- | --- | --- | --- | --- | --- | --- | --- | --- | --- | --- | --- | --- | --- |
|  | (m^2^g^-1^) | (%) |  | C | H | N | O | S |  | H/C | O/C |  | f_a_ | f_b_ | f_c_ |
| 10.4 | 3.77 | 26.99 |  | 49.74 | 2.61 | 2.31 | 18.35 | 1.9 |  | 0.63 | 0.28 |  | 0.83 | 0.084 | 0.039 |

f_a_: fraction of corrected aromaticity; f_b_: phenolics or phenolic esters; f_c_: carbons bonded to oxygen.

**Table S3** Electron equivalents consumed for each microbial reduction processes in different treatments.

| Treatments^a^ | Electron equivalents（µmol） consumed for microbial reductions of | | | | Total electron equivalents needed (µmol) |
| --- | --- | --- | --- | --- | --- |
|  | Ferric iron reduction | Sulfate reduction | Dechlorination (1-Cl) | Methanogenesis |  |
|  |  |  |  |  |  |
| CK | 1217.18c | 165.17b | 1.55a | 1641.86a | 3033.52b |
| A | 2096.53a | 89.67bc | 0.33cd | 1615.85a | 3804.04a |
| B | 1725.97ab | 501.29a | 0.79b | 1634.22a | 3866.25a |
| MCK | 1198.43c | 72.27c | 0.15de | 34.56c | 1306.14d |
| MA | 1427.53bc | 60.11c | 0.00e | 0.00c | 1487.64d |
| MB | 1031.75c | 98.95bc | 0.4c | 1351.80b | 2485.37c |

ANOVA analysis, p<0.05

^a^Abbreviations for each treatment are the same as Fig. 2. ^b^Electron equivalents for PCP dechlorination were rather small comparing with other reduction processes, so the degradation of 2,3,4,5-TeCP and 3,4,5-TCP were not calculated.
